# Azithromycin non-susceptible *Shigella* circulating in Israel, 2014–2016

**DOI:** 10.1371/journal.pone.0221458

**Published:** 2019-10-18

**Authors:** Analía V. Ezernitchi, Elizabeta Sirotkin, Dana Danino, Vered Agmon, Lea Valinsky, Assaf Rokney

**Affiliations:** 1 Government Central Laboratories, Ministry of Health, Jerusalem, Israel; 2 Pediatric Infectious Disease Unit, Soroka University Medical Center, Beer Sheva, Israel; 3 Faculty of Health Sciences, Ben-Gurion University of the Negev, Beer Sheva, Israel; Universidad Nacional de la Plata, ARGENTINA

## Abstract

*Shigella* species remains a major diarrhoeagenic agent, affecting mostly children, with global high incidence and high mortality rate specially in developing areas. Although azithromycin is recommended for treatment of shigellosis, there are currently no CLSI susceptibility breakpoints, accordingly no routine antimicrobial susceptibility test is performed in the clinical laboratory. The purpose of this study was to estimate the prevalence, resistance profile and molecular epidemiology of azithromycin non-susceptible *Shigella* strains in Israel during a three year period. *Shigella* isolates (n = 1,170) referred to the National Reference Center during 2014–2016, were included in this study. Serotyping was performed by slide agglutination. Resistance genes, *mph*(A) and *erm*(B), were identified by PCR and the phenotype profile was determined by broth microdilution (BMD). Genetic relatedness was assessed by wgMLST. Decreased susceptibility to azithromycin (DSA) phenotype and genotype were detected in various *Shigella* species and serotypes related to diverse genetic backgrounds and antimicrobial profiles: 6% (26/423) of *Shigella flexneri* and 2% (16/747) of *Shigella sonnei* displayed DSA (MIC16 mg/L). Correlation of this phenotype with the presence of *mph*(A) and *erm*(B) genes was confirmed. All DSA-strains displayed resistance to ≥3 different antimicrobial classes. Among DSA-strains, 14% were resistant to quinolones and 5% displayed resistance to ceftriaxone. Most of these strains (32/42) were isolated from children in the southern and central regions of Israel. Clonality and significant relatedness was confirmed by PFGE and wgMLST. The presence of macrolide resistance genes among the different species and lineages reflects the transmissible nature of these genes. The emergence of DSA-*Shigella* reinforces the necessity to establish clinical breakpoints that would warrant routine testing, reporting and surveillance for this drug of choice.

## Introduction

*Shigella* species remain an infectious agent of public concern, affecting mostly children, with high incidence in developing areas as well as in industrialized countries. In 1999 the global burden of shigellosis was estimated as 164.7 million cases [1]. A more recent study established *Shigella* as the second leading cause of diarrheal death among children globally (54,900 deaths, 2015) [2, 3]. Incidence and serotypes vary among industrial and developed regions. Shigellosis incidence in Israel is among the highest in the industrialized world, ranging from 70 to 100 cases / 100,000 population per year. Incidence peaks are observed biennially in a cyclic pattern, and the burden is mostly attributed to *Shigella sonnei* (90%) [4].

Although shigellosis is a self-limiting disease, antibiotic therapy is effective for clinical management and can prevent major complications. It also contributes to shorten the shedding period, which is of relevance in terms of prevention of this extremely infective disease [5].

As ampicillin and trimethoprim-sulfamethoxazole are not appropriate for the treatment of shigellosis [6], the WHO recommendation for first and second line therapy are ciprofloxacin and ceftriaxone respectively. However, the emergence of ESBL-producing and quinolone resistant strains highlight the relevance of azithromycin as the drug of choice especially for children, usually empirically administered. Although azithromycin is recommended worldwide for treatment of shigellosis [7], there are currently no CLSI or EUCAST susceptibility breakpoints. Recently, the emergence of *Shigella* with decreased susceptibility to azithromycin (DSA) has been reported globally [8–13]. DSA-*Shigella* strains displayed MIC ≥16 mg/L. In many of these reports a phenotypic-genotypic correlation was established. Two macrolide resistance genes *mph*(A) and *erm*(B) confer reduced susceptibility to azithromycin by two different mechanisms of resistance, drug inactivation and target-site modification, respectively [14, 15]. Information regarding local resistance rates is essential when delineating policies concerning antibiotic therapy. The Israel National Reference Center for *Shigella* (NRCS) performs routine surveillance of antimicrobial resistance on isolates received from all hospitals and community health centers. In this study we aimed to assess the prevalence, resistance mechanisms and molecular epidemiology of DSA-*Shigella* in Israel.

## Materials and methods

### Bacterial strains

All *S*. *flexneri* and a sample of *S*. *sonnei* strains referred to the NRCS during a three-year period (2014–2016) were included in this study. During the study period 6,712 *S*. *sonnei* were identified at the NRCS and a sample of strains (n = 747, 11%) from all sending laboratories, isolated during all months of the study period were analyzed. Genus confirmation and species identification was performed by biochemical conventional tests [16], and serotyping by slide agglutination (Denka Seiken, Japan).

### Polymerase chain reaction (PCR)

Detection of *mph*(A) and *erm*(B) genes was performed by PCR as previously described [17]. Briefly, DNA was extracted by lysing the bacteria at 95°C for 15 minutes. The primers used to detect *mph*(A) and *erm*(B) genes were: *mph*(A)(F) GTG AGG AGG AGC TTC GCG AG *mph*(A) (R) TGC CGC AGG ACT CGG AGG TC and *erm*(B)(F) GAA AAG GTA CTC AAC CAA ATA *erm*(B) (R) AGT AAC GGT ACT TAA ATT GTT TAC respectively [18]. DNA lysate (1μl) was incorporated into PCR reaction mixture (20μl) containing 1.5mM MgCl_2_ (ReddyMix PCR Master, Thermo Scientific). Thermal cycling reaction was performed using the following conditions: 93°C for 3 min, followed by 35 cycles of 93°C for 1 min, 52°C for 1 min, 72°C for 1 min; a final elongation step was performed at 72°C for 5 min. Sequencing of PCR results was performed at the Center for Genomic Technologies, Institute of Life Science, The Hebrew University of Jerusalem (Jerusalem, Israel). Sequence analysis by BLAST was performed using Bionumerics version 7.6. software (Applied maths).

### Antimicrobial susceptibility test (AST)

Susceptibility to 14 antimicrobial agents was determined by broth microdilution (Sensititre, Trek diagnostics) according to CLSI breakpoints where available and manufacturer instructions. Agents tested were: amoxicillin/clavulanic acid (AMC), ampicillin (AMP), azithromycin (AZM), cefoxitin (FOX), ceftriaxone (CRO), chloramphenicol (CHL), ciprofloxacin (CIP), gentamicin (GEN), meropenem (MER), nalidixic acid (NAL), sulfisoxazole (FIS), streptomycin (STR), tetracycline (TET), trimethoprim/ sulfamethoxazole (SXT).

In the absence of CLSI breakpoints for azithromycin and streptomycin, interpretative criteria were applied according to NARMS [19]. DSA- *Shigella* was defined as *S*. *sonnei* and *S*. *flexneri* displaying MIC ≥32 mg/L and ≥16 mg/L respectively. Streptomycin resistant strains were defined as those displaying MIC ≥32 mg/L.

### Pulsed-field gel electrophoresis (PFGE)

Agarose-embedded (Lonza, USA) DNA was digested with XbaI (Fermentas, Lithuania) followed by gel electrophoresis in the CHEF MAPPER (Bio-rad) system. Electrophoresis conditions were 14°C, 0.5×Tris-borate-EDTA buffer (Sigma, Switzerland), initial pulse 2.2 s, final pulse 54.2 s, 6V, 18 h. Salmonella Braenderup H9812 restricted with *Xba*I (Fermentas, Lithuania) was used as a marker. PFGE restriction patterns were analyzed by the BioNumerics 7.6 software (Applied Maths). The pulsotypes were compared using the band-based DICE similarity coefficient with 1% optimization and tolerance. The un-weighted pair group method with arithmetic mean (UPGMA) algorithm was used for cluster analysis

### Whole genome sequence (WGS)

A sample of DSA-*Shigella* strains (19 *S*. *flexneri*, 12 *S*. *sonnei*) was subjected to Genomic DNA extraction using the QiaSymphony platform (Qiagen). Libraries were built with the Nextera XT kit according to manufacturer protocol and sequenced 2X250bp at X100 coverage using the MiSeq or HiSeq platforms (Illumina). Raw sequence reads were deposited in the National Center for Biotechnology Information (BioProject PRJNA527971 and PRJNA527970, accession numbers are listed in S1 Table).

### Genome assembly and wgMLST

Paired-end 250 bp reads were de-novo assembled by SPAdes on the BioNumerics calculation engine. The raw reads and SPAdes assemblies were analyzed with the BioNumerics wgMLST pipeline. The genomes were compared by minimum spanning tree (MST) based on allelic calls. Multi locus sequence types were predicted from the reads and assemblies. Reads of DSA *Shigella* publicly available and included in published studies were used in reference and comparison [20–26] (S2 Table).

### Phylogeny and comparative genomics

In order to identify the nearest reference genome, reads were analyzed by the NCBI Pathogen Detection pipeline (https://www.ncbi.nlm.nih.gov/pathogens/). The local DSA strains were assigned SNP cluster with <50 SNP distance, and antimicrobial resistance genes were identified with the NCBI AMR Finder tool. A list of clusters with available accession numbers, AMR genotype and location is detailed in S4 Table (*S*. *flexneri*) and S5 Table (*S*. *sonnei*). The clustered sequences were analyzed in Enterobase and a wgMLST based grape tree was generated for each serogroup.

## Results

**Fig 1:**
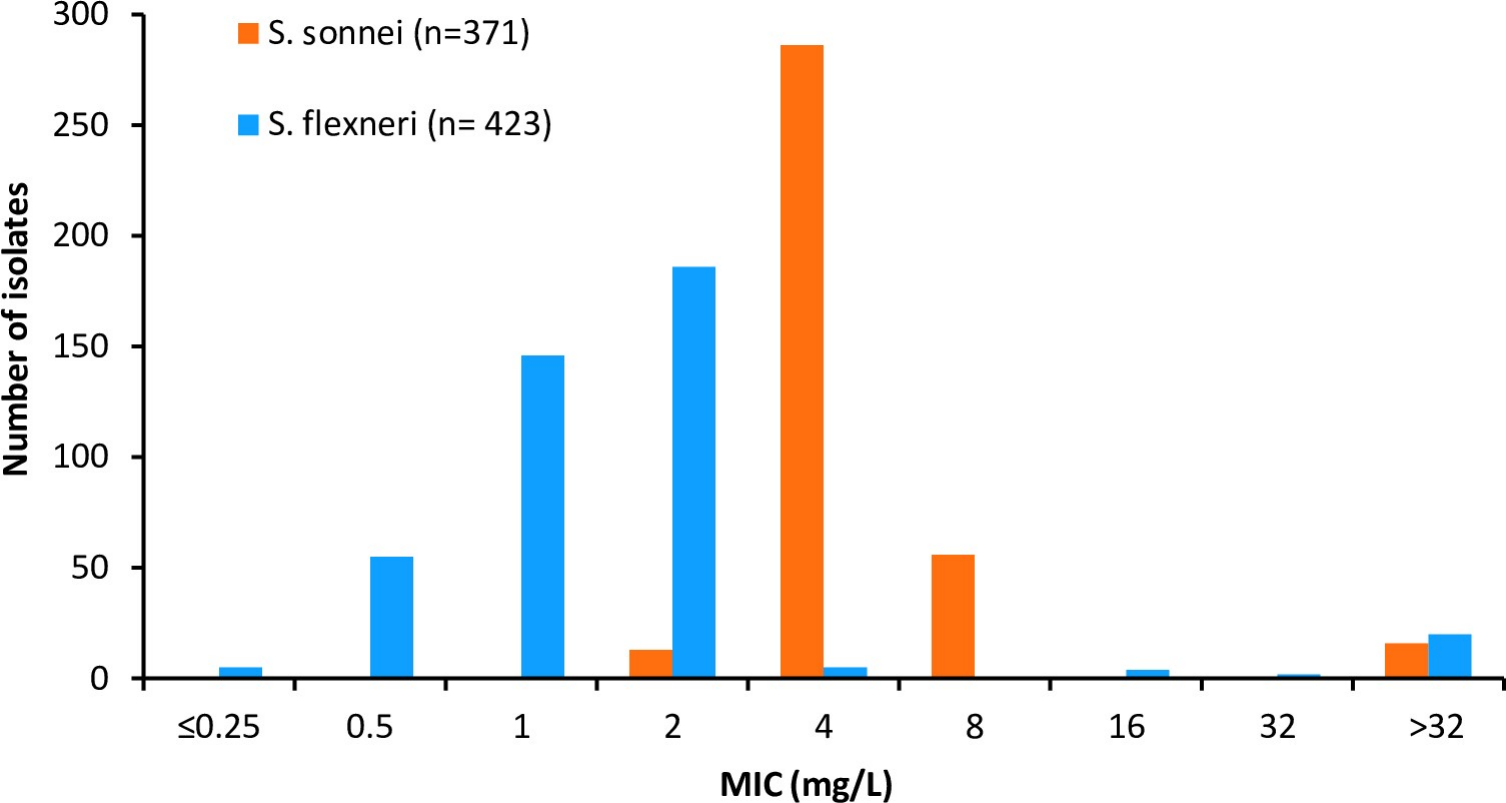
Distribution of azithromycin minimum inhibitory concentrations (MICs) for *S*. *flexneri* isolates (n = 423) and *S*. *sonnei* (n = 371).

In vitro susceptibility testing was performed on 423 *S*.*flexneri* strains (Fig 1). A total of 26 strains (6.1%) displayed decreased susceptibility to azithromycin (DSA). The MIC value obtained for DSA strains was higher or equal to 16 mg/L (MIC_90_ was >32 mg/L) while the rest of the 423 strains displayed MIC of 4 mg/L or lower. DSA phenotype correlated with the presence of macrolide resistance genes. As shown in Table 1, the *mph*(A) gene was detected alone or together with *erm*(B) gene in 25 strains. One strain was found to harbour the *erm*(B) gene alone. All *S*. *sonnei* strains included in this study (n = 747) were screened by PCR (Table 1), and a subset of 371 strains, including all the strains carrying macrolide resistance genes, were tested by BMD method (Table 1 and Fig 1). In total, 16 out of 747 (2.1%) *S*. *sonnei* isolates were found to display AZM MIC value >32, all harboured *mph*(A) gene, while the *erm*(B) gene was detected in a single strain isolated during 2016 (S1 Table).

**Table 1:** Prevalence of macrolide resistant genes in *S*.*flexneri* and *S*. *sonnei*.

**year**	**No. of *S*.*sonnei* strains tested**	***erm*(B)- *mph*(A)+** **% (n)**	***erm*(B)+*mph*(A)-** **% (n)**	***erm*(B)+*mph*(A)+** **% (n)**	**Total PCR+ strains**^**a**^ **% (n)**
2014	203	2% (4)	0% (0)	0% (0)	2%(4)
2015	200	1% (2)	0% (0)	0% (0)	1% (2)
2016	344	2.6% (9)	0% (0)	0.3% (1)	2.9% (10)
**Total**	**747**	**2% (15)**	**0% (0)**	0.3% (1)	**2.1% (16)**
**year**	**No. of *S*.*flexneri* strains tested**	***erm*(B)- *mph*(A)+** **% (n)**	***erm*(B)+*mph*(A)-** **% (n)**	***erm*(B)+*mph*(A)+** **% (n)**	**Total PCR+ strains**^**a**^ **% (n)**
2014	161	4.3% (7)	0% (0)	2.4% (4)	6.8% (11)
2015	132	4.5% (6)	0% (0)	0.8% (1)	5.7% (7)
2016	130	3.8% (5)	0.7% (1)	1.5% (2)	6.1% (8)
**Total**	**423**	**4.3% (18)**	**0.2% (1)**	**1.6% (7)**	**6.1%(26)**

^a^the total PCR+ strains includes the total number of strains positive for one or both resistance genes (*mph*(A) and *erm*(B)) detected by PCR.

DSA-*S*. *flexneri* included different serotypes: 1b (n = 12), 6 (n = 5), 2a (n = 5), 1a (n = 1), 2b (n = 1), 3a (n = 1) and *S*. *flexneri* variant Y (n = 1). *S*. *sonnei* and *S*. *flexneri* isolates displayed diverse antimicrobial resistance patterns as shown in Table 2. Resistance to ampicillin was displayed by 93% of the isolates. Multidrug resistance phenotype, resistance to ≥3 classes of antimicrobial agents, was identified among all DSA stains (100%). Extensive drug resistant phenotype, resistance to ≥5 classes of antimicrobial agents, was identified in 13 out of 42 (31%) DSA isolates. Notably, resistance to ceftriaxone, together with ampicillin and streptomycin, was observed in two strains (*S*. *flexneri* serotype 1b) further confirmed as ESBL-producing strains. Resistance to quinolones (nalidixic acid) was observed in 6 strains from different serotypes, including two strains displaying resistance to ciprofloxacin. None of the isolates included in this study displayed resistance to carbapenems.

**Table 2:** DSA *S*. *flexneri* and *S*. *sonnei* antimicrobial resistance profiles.

Antimicrobial resistance profile	*S*. *flexneri*							*S*. *sonnei*
	1a	1b	2a	2b	3a	6	Variant Y	
AMC AMP AZM FOX CIP NAL STR FIS TET SXT		1						
AMC AMP AZM FOX CIP NAL STR TET			1					
AMP AZM CHL STR FIS SXT			1					
AMP AZM CHL STR FIS TET SXT			3	1	1	1		
AMP AZM CHL STR TET							1	
AMP AZM CRO STR		2						
AMP AZM NAL STR FIS TET SXT	1					2		
AMP AZM STR		8						12
AMP AZM STR FIS SXT		1						3
AZM FIS TET SXT						2		
AZM NAL STR FIS SXT								1

AMC, amoxicillin/ clavulanic acid; AMP, ampicillin; AZM, azithromycin; FOX, cefoxitin; CRO, ceftriaxone; CHL, chloramphenicol; CIP, ciprofloxacin; NAL, nalidixic acid; FIS, sulfisoxazole; STR, streptomycin; TET, tetracycline; SXT, trimethoprim/ sulfamethoxazole.

**Fig 2:**
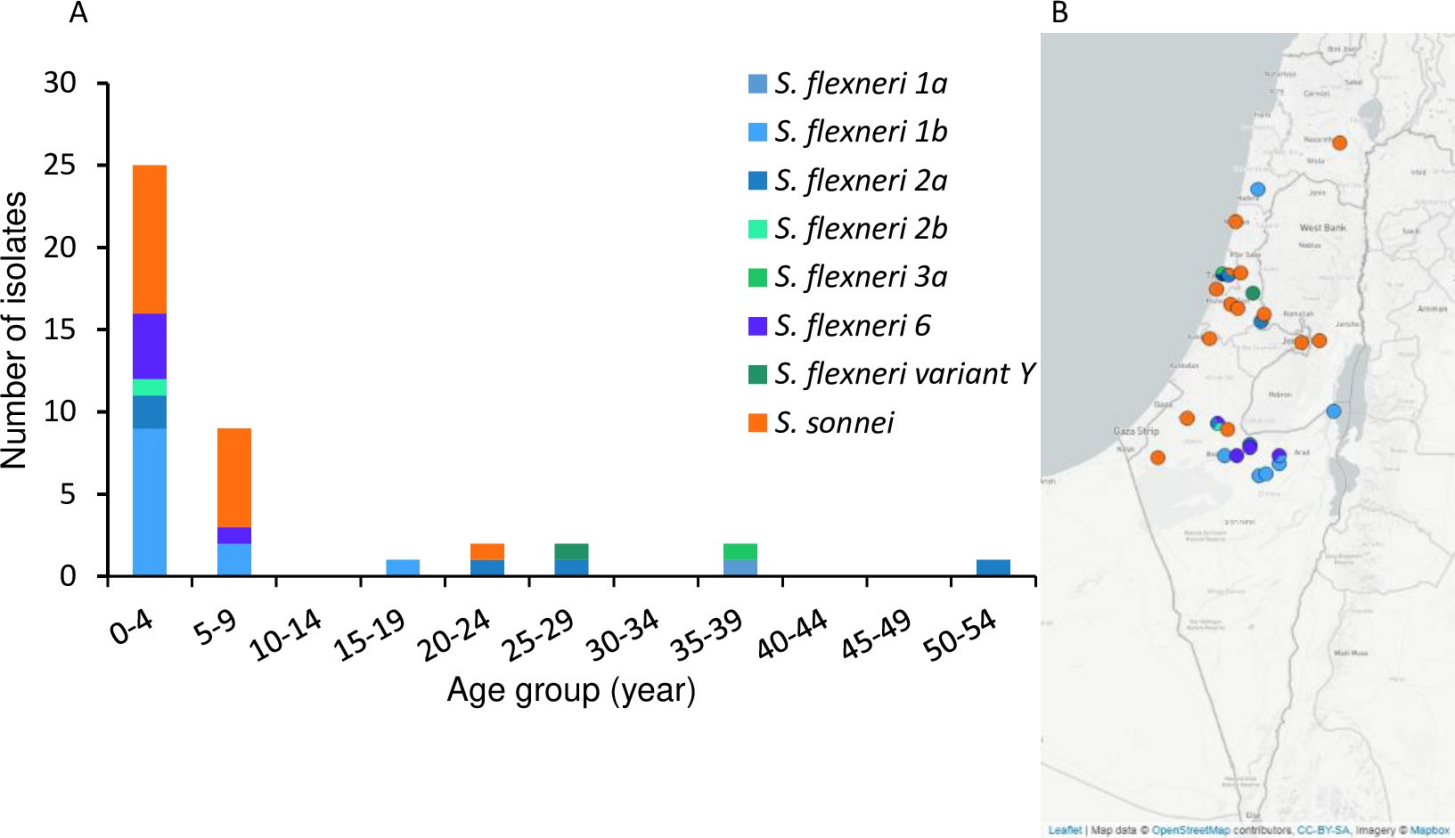
Geographical and age distribution of DSA-*S*. *flexneri* (n = 26) and DSA-*S*. *sonnei* (n = 16). (A) Distribution of DSA-*S*.*sonnei* (n = 16) and DSA-S. *flexneri* (n = 26) strains according to age group. (B) Geographical distribution (n = 42). Map was generated with Microreact [27].

Geographic and age distribution analysis of DSA-*S*. *flexneri* strains showed that 19 out of 26 (73%) were isolated in the southern region of Israel, and corresponded to ages ranging from newborn to 9 years of age, except for one strain isolated from a 17 years old male (Fig 2A and Fig 2B). Most of these, (17/19), were isolated within the Bedouin population of southern Israel. One strain was isolated in the north, and the remaining 6 strains, were exclusively isolated from males, age ranged from 22 to 54 years living in the center of Israel.

DSA-*S*. *sonnei* strains were isolated mainly from children under 9 years of age (15 out of 16), only one strain corresponded to a young adult from the central west region of Israel (Fig 2). 69% of DSA-*S*. *sonnei* were isolated in the central region. Complete description of demographic distribution with isolation date, serotype, age and gender is shown in supporting information (S1 Table).

**Fig 3:**
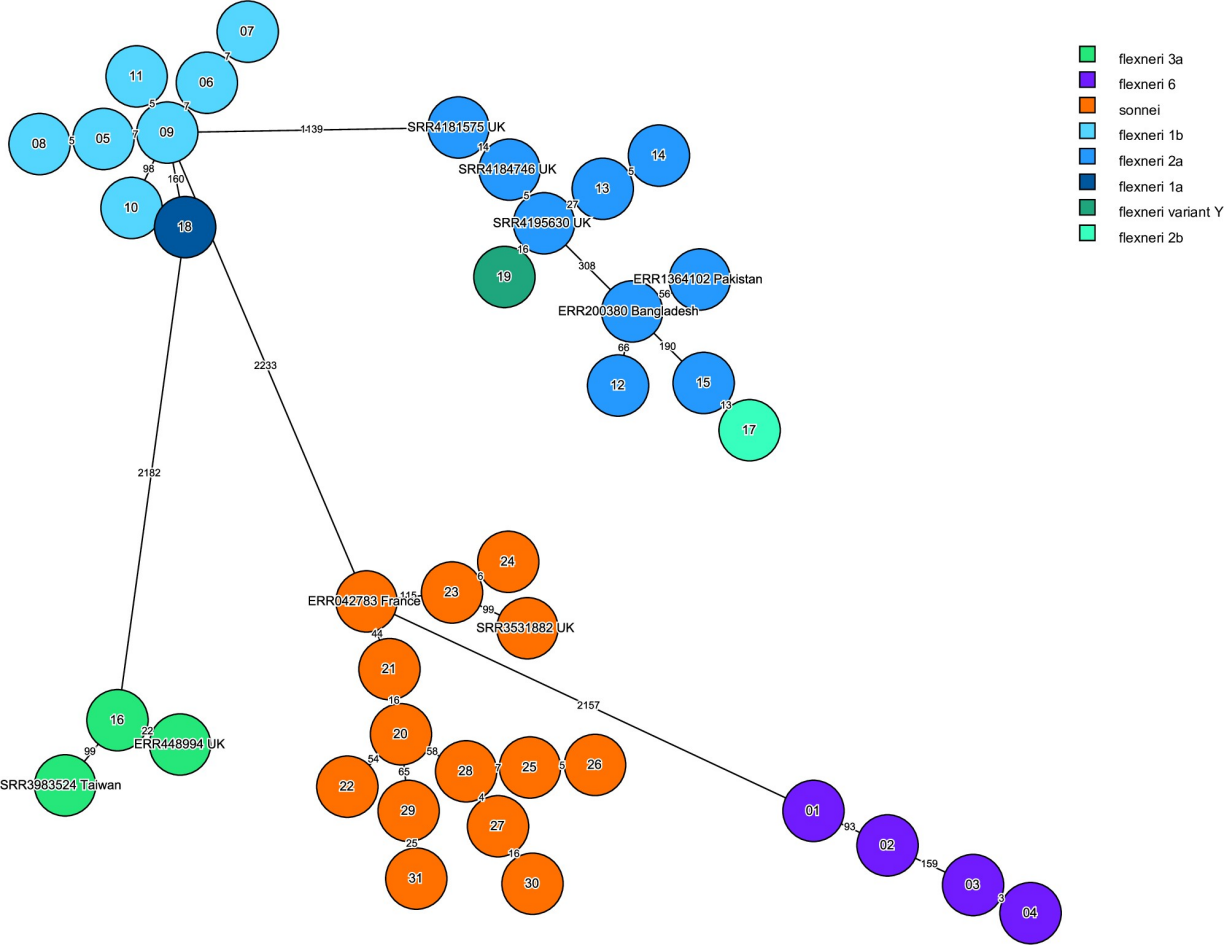
wgMLST of DSA-*Shigella* isolates, (2014–2016). A sample of 31 DSA-strains, representing all the serogroups and PFGE profiles obtained for the 42 DSA-strains, was analyzed by wgMLST. Each circle represents one strain. Allelic differences between two strains are indicated by the numbers on the lines connecting the strains (branches). Strain numbers are indicated at the center of each circle. For Israeli strains only the last 2 digits are displayed. For international context, strains accession numbers (SRA, ENA) are indicated (further information in S2 Table).

Relatedness among DSA-*Shigella* isolates was assessed by PFGE (S1 Fig.) and wgMLST (Fig 3).

WGS analysis confirmed clonality (≤5 allele difference) within the *S*. *flexneri* serotypes (1b, 2a, 6) and the *S*. *sonnei* species, indicating a possible common source of transmission (Fig 3). *S*. *flexneri* 1b clonal strains were from cases in southern Israel, *S*. *flexneri* 2a cases in central Israel, *S*. *flexneri* 6 in southern and central Israel. The WGS clonality within *S*. *sonnei* was consistent with PFGE results (S1 Fig), clusters were distributed in the south, north and center regions of the country.

We compared our sequences with previously published global strains (Fig 3). The Israeli strains were genetically related (20–115 alleles) with globally reported strains from Bangladesh (*S*. *flexneri* 2a), UK (*S*. *flexneri 2a*, *S*. *flexneri 3a* and *S*. *sonnei*), France (*S*. *sonnei*) and Pakistan (*S*. *flexneri 2a*).The only *S*. *flexneri* 3a strain found in this study was sequenced and compared to previously reported pandemic strain from a study conducted in UK (ENA accession no. ERR448994) and to a DSA- *Shigella flexneri* 3a reported in Taiwan (NCBI accession no. SRR3983524) [23, 24]. The distance calculated by wgMLST analysis was 22 and 99 alleles between respective strains.

**Fig 4:**
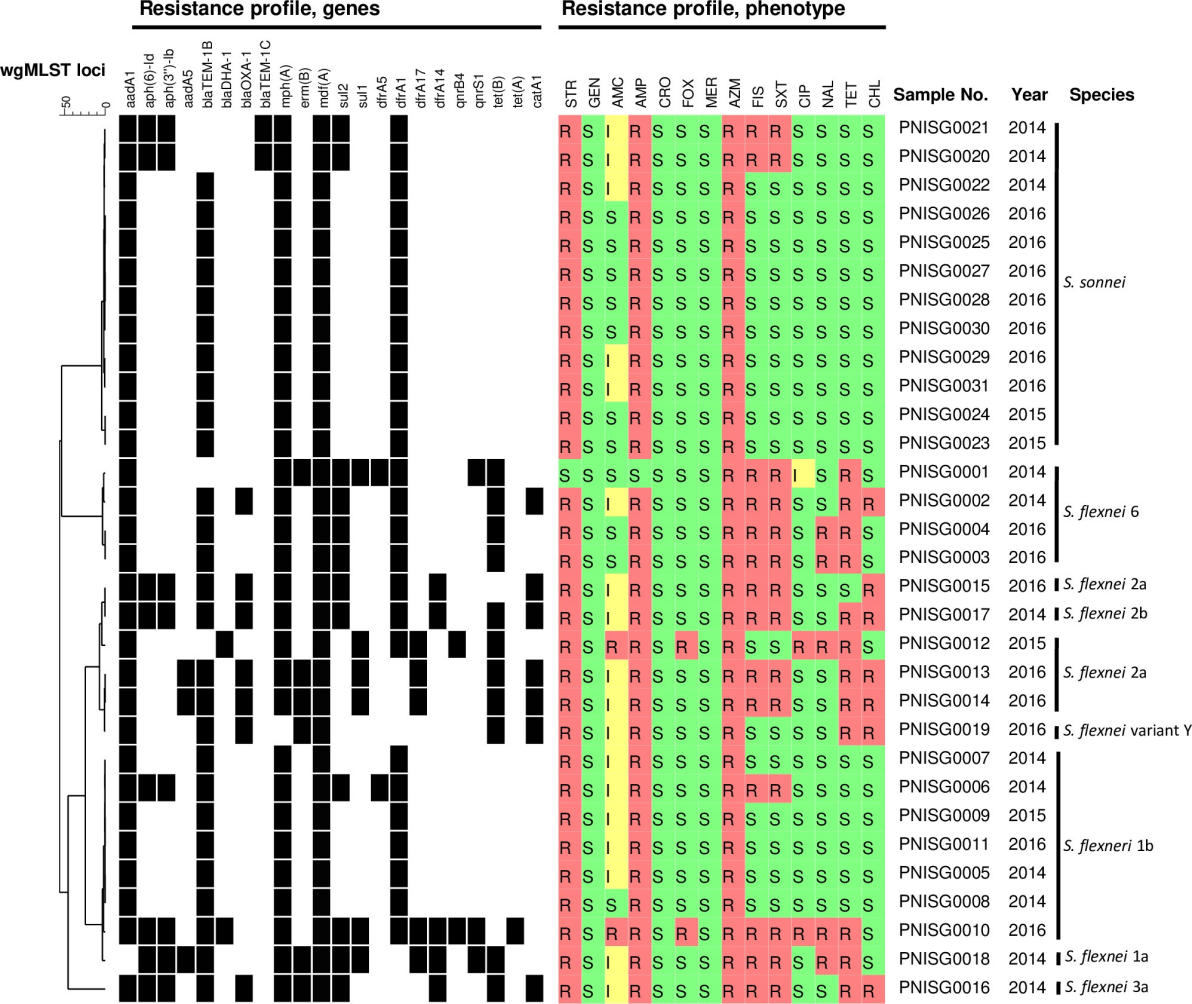
Genotype and phenotype AMR profile correlation. Resistance genes (n = 22) were identified using BioNumerics tool, black cell indicate the presence of gene. Phenotypic results (14 antimicrobial agents tested) are expressed by S (susceptible, green cell), I (intermediate, yellow cell) and R (resistant, red cell).

Comparison between antimicrobial resistance genes and phenotype was performed for 31 DSA-strains subjected to WGS (Fig 4). Genotype prediction for susceptibility was correct for 29 out of 31 isolates (431 tests out of 434, 99.3%). Disagreement was found for two isolates: one isolate (PNISG0001) displayed susceptibility to streptomycin and the second isolate (PNISG0012) displayed susceptibility to sulfisoxazole and trimethoprim/sulfamethoxazole, this phenotype was not correlated with the presence of genes associated with resistance to aminoglycosides, trimethoprim and sulphonamides.

For further investigation and contextualization of strains, sequences uploaded to NCBI were compared with all available *Shigella* sequences in NCBI database. Using NCBI's Pathogen, the nearest international sequences were identified for each of the Israeli DSA strains. Each cluster was assigned with a cluster number (SNP number). Resume of findings is shown in S3 Table. All Israeli DSA-strains analyzed by WGS (n = 31) together with nearest international strains found within SNP clusters were used to generate wgMLST grape trees with Enterobase [28].

**Fig 5:**
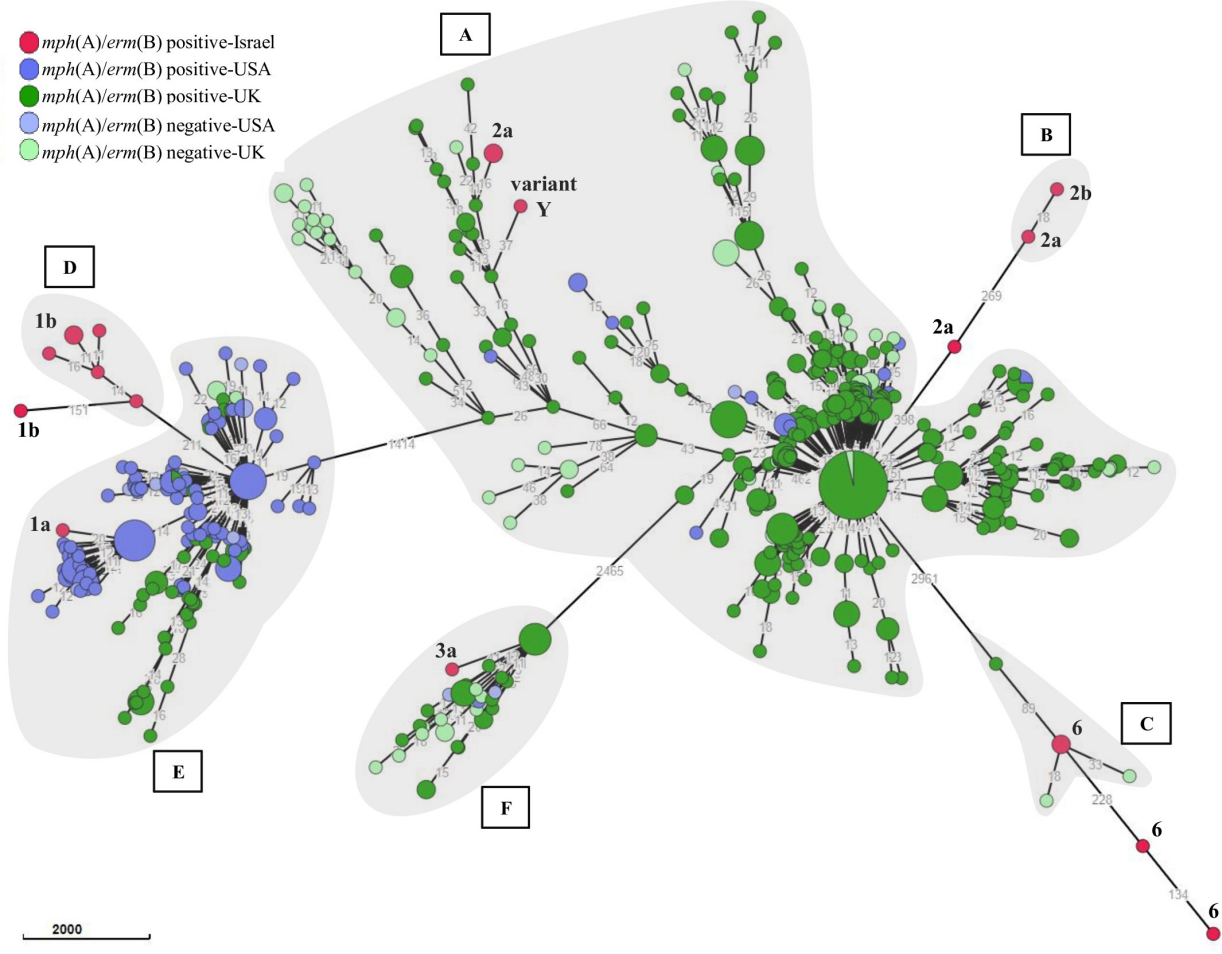
wgMLST of *S*. *flexneri* strains. wgMLST based minimum spanning tree of 733 strains is shown. Clusters of strains, as identified in Pathogen detection are shaded (grey) and denoted by capital letters each representing a SNP cluster number: PDS00006028.119 (A), PDS000044321.1 (B), PDS000020403.6 (C), PDS000044322.1 (D), PDS00007822.141 (E) and PDS00006023.31 (F). The presence or absence of macrolide resistance genes and country are indicated.

Seven DSA-*S*. *flexneri* local strains (*S*. *flexneri* 3a, *S*. *flexneri* 6, *S*. *flexneri* 2a, *S*. *flexneri* variant Y and *S*. *flexneri* 1a) were linked to international strains distributed in four different Pathogen Detection clusters (PDS00006023.31, PDS000020403.6, PDS00006028.119, PDS00007822.141). Macrolide resistance genes, *mph*(A) and *erm*(B), were not evenly distributed in all clusters. The rest of the local strains (12 out of 19) did not cluster with any international strain, these included *S*. *flexneri* 2a, *S*. *flexneri* 2b, *S*. *flexneri* 1b and *S*. *flexneri* 6 (Fig 5).

**Fig 6:**
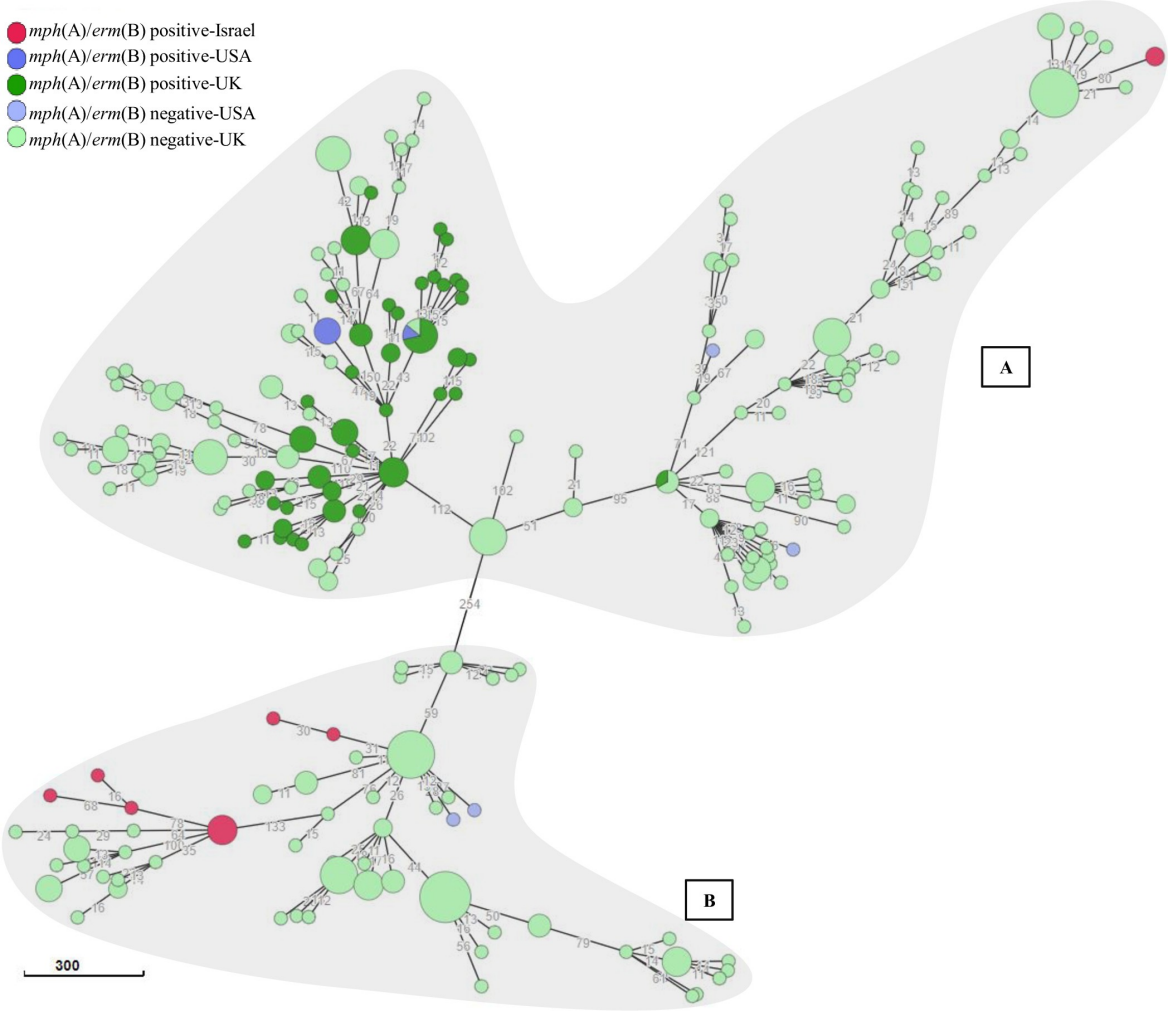
wgMLST of *S*. *sonnei* strains. wgMLST based minimum spanning tree of 400 strains is shown. Clusters of strains, as identified in Pathogen detection are shaded (grey) and SNP cluster number indicated by capital letters: PDS000035344.47 (A) and PDS000036978.7 (B). Presence or absence of macrolide resistance genes and country are indicated.

All DSA-*S*. *sonnei* strains (n = 12) were distributed in two clusters (Fig 6). Both clusters included Israeli and international strains. Notably, 10 local strains clustered only with azithromycin susceptible international strains (Fig 6, cluster B).

## Discussion

This is the first report showing data on azithromycin resistance of *Shigella* sp. in Israel. In this retrospective study, a stable rate of azithromycin resistance was observed within *S*. *sonnei* as well as *S*. *flexneri* groups, over the 3 years that isolates were obtained.

Consistent with other reports [12, 19, 29] and the cutoff values published by CLSI [30], lower MIC values were obtained for susceptible *S*. *flexneri* than for susceptible *S*. *sonnei*. Macrolide resistance genes were detected only among those strains displaying MIC higher or equal to 16 mg/L and 32 mg/L in *S*. *flexneri* and *S*. *sonnei* respectively. The *mph*(A) gene was found to be more prevalent than *erm*(B) among DSA-*Shigella* strains.

DSA-*Shigella* strains were well disseminated among different regions and diverse population (ethnicity, age, gender). Clonality could be established to some extent; and some strains were identified as part of an outbreak (e.g. *S*. *flexneri* 1b in the south and *S*. *flexneri* 2a in the center region).

The presence of *mph*(A) and *erm*(B) genes in different *Shigella* serogroups supports the probability of different gene acquisition sources (including gene transfer). Plasmid characterization was not performed in this study.

Contextualization analysis using Pathogen detection and Enterobase indicated that 12 out of 31 isolates did not match any global cluster, representing novel DSA-*Shigella* strains. Clustering of DSA-*S*. *sonnei* with only azithromycin susceptible strains, reinforced the hypothesis of local macrolide resistance genes acquisition as a plausible explanation for the expansion of DSA-phenotype among distinct serogroups in Israel. Seven *S*. *flexneri* 1b were divided in two clusters one of 6 strains, included only Israeli isolates, and the other, only one strain which did not clustered at all, consistent with wgMLST analysis in Fig 3. This singular *S*. *flexneri* 1b strain differed from the rest also in terms of AMR profile and the isolation region (isolated in the north and not in the southern region like the others). Notably, the only DSA-*S*. *flexneri* 1a reported in this study was found within a cluster composed mainly by strains carrying macrolide resistant genes isolated in USA (PDS00007822.141). A limitation of the comparison analysis between local and international strains, conducted in this study, is that though clusters extracted from Pathogen detection included strains from different world regions (UK, USA, Australia, Belgium and France), the Enterobase analysis used only a limited number of available sequences. So only strains from USA and UK are visualized in final tree. Further high-resolution comparison of local DSA-*Shigella* strains to globally reported strains is essential in order to clarify the possible source of these strains in Israel and the mode of transmission.

Sexual transmission, as previously reported [23, 31], could be tentative hypothesized for DSA-*S*.*flexneri* 2a: a cluster of 3 strains isolated during a period of five months from adult men living in the same region. This cluster was characterized by carrying *erm*(B) gene, differing from the rest of this serotype group which harbored *mph*(A) alone. However, this assumption could not be confirmed since no data regarding sexual activities was collected. Two of these strains were analyzed by WGS, and found to be genetically related to UK strains harboring macrolide resistance genes (Fig 3 SRR4195630 and Fig 5 cluster PDS00006028.119).

Dissimilar from others reports [8, 12, 31–33], in this study the main affected population (80%) was children under 10 years of age. This is in line with local epidemiological data, in Israel above 80% of shigellosis cases occur in 0 to 15 age group range [34]. All 6 DSA strains resistant to quinolones and the DSA-ESBL strains were acquired by children less than 4 years of age. Despite azithromycin being one of the drugs of choice for the treatment of shigellosis, especially in children, there are still no guidelines for interpretation of susceptibility tests. Thus, *Shigella* spp. susceptibility to azithromycin is not currently tested in clinical microbiological laboratories. In Israel, the emergence of quinolone and fluoroquinolone resistant strains was first observed in 2013. During 2016, 44% of *S*. *flexneri* strains were resistant to nalidixic acid and 10% to ciprofloxacin [35]. Cases of shigellosis with isolates displaying resistance to ceftriaxone or ciprofloxacin would be treated with azithromycin. In this scenario, decreased susceptibility to azithromycin would lead to therapy failure. Indeed, in 2014 DSA-*Shigella* were isolated from single patient within a 28-day period, indicative of a possible treatment failure, persistent shedding or reinfection. Partial access to medical data revealed that azithromycin was given as therapy for the treatment of the first shigellosis episode (S1 Table).

Establishment of clinical breakpoint and susceptibility test implementation in the clinical laboratory are a major public health priority. Azithromycin is a broad spectrum antibiotic usually given as therapy for many different types of bacterial infections [36]. Acquisition of resistance genes by *Shigella*, which is endemic and widely distributed across Israel, could facilitate the dissemination of transferrable macrolide resistant genes within *Shigella* and across species.

Thus, routine surveillance of *Shigella* species and antimicrobial profiles is crucial to understand modes of transmission and sources of infection. The information presented here should be used for the future establishment of guidelines and the management of antimicrobial therapy in children as well as in adults.

## Supporting information

S1 Table DSA-*Shigella* strains recovered in Israel during 2014–2016. (XLSX)

S2 Table Reference sequences of publically available DSA-*Shigella*. (XLSX)

S3 Table Resume of results using Pathogen detection (NCBI). (XLSX)

S4 Table *S*. *flexneri* strains obtained from Pathogen detection searched in Enterobase. (XLSX)

S5 Table *S*. *sonnei* strains obtained from Pathogen detection searched in Enterobase. (XLSX)

S1 Fig Genetic relatedness among DSA-*Shigella* isolates (2014–2016). Pulsed-field gel electrophoresis (PFGE) results showing *Xba*I digested DNA patterns of DSA-*S*. *flexneri Shigella sonnei*.
(TIF)
