# Supplementary figures and images for: Azithromycin non-susceptible Shigella circulating in Israel, 2014–2016
Source: PLoS One. 2019 Oct 18;14(10):e0221458. doi: 10.1371/journal.pone.0221458 (PMC6799884; doi:10.1371/journal.pone.0221458)

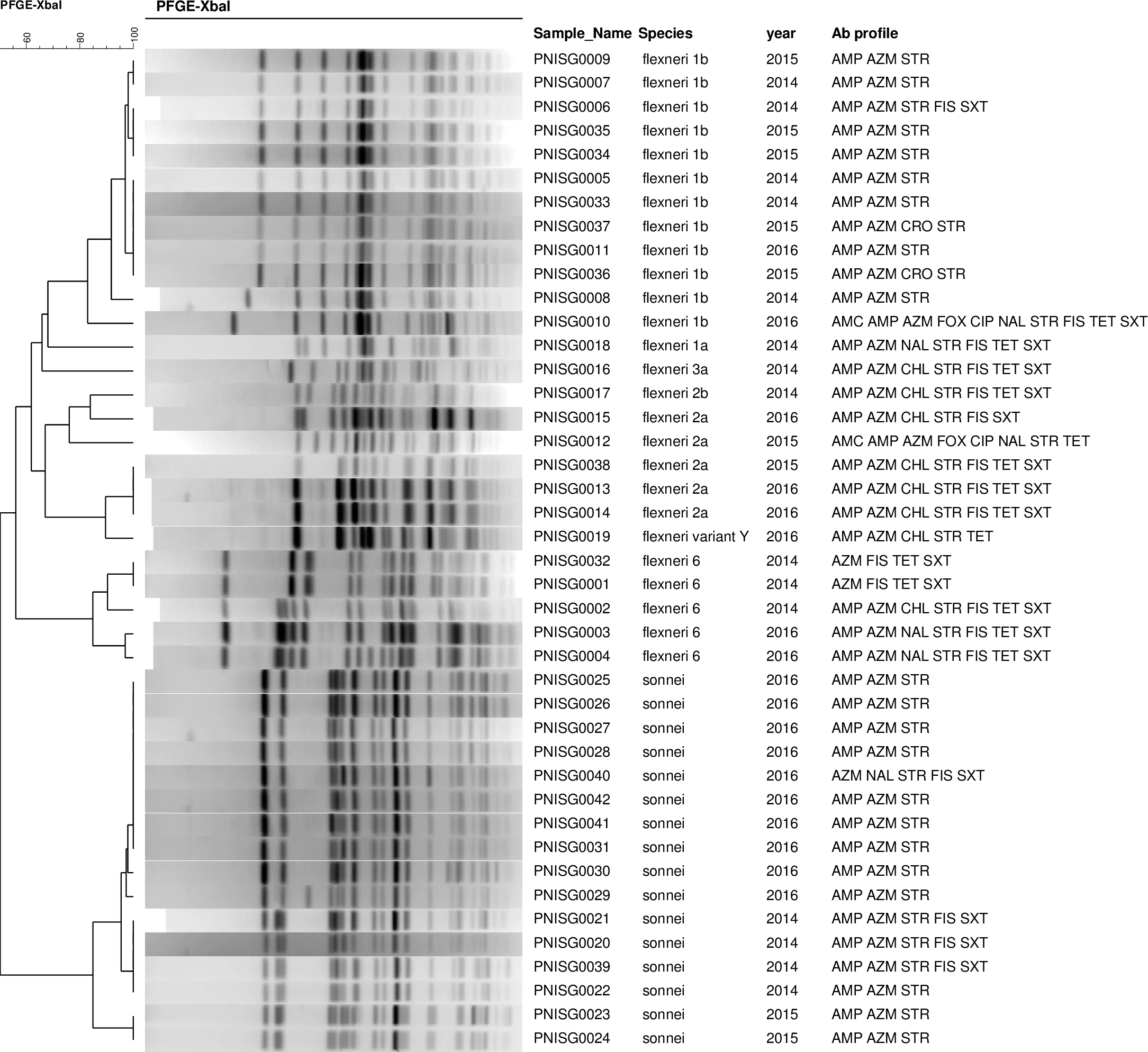

Supplement: S1 Fig — Pulsed-field gel electrophoresis (PFGE) results showing XbaI digested DNA patterns of DSA-S. flexneri Shigella sonnei. (TIF) [file pone.0221458.s006.tif]
